# Metabolic interplay of SCFA’s in the gut and oral microbiome: a link to health and disease

**DOI:** 10.3389/froh.2025.1646382

**Published:** 2025-08-25

**Authors:** Nireeksha, Alexander Maniangat Luke, Suchetha Kumari N, Mithra N. Hegde, Nishmitha N. Hegde

**Affiliations:** ^1^ Conservative Dentistry and Endodontics, AB Shetty Memorial Institute of Dental Sciences, Nitte (deemed to be) University, Mangalore, India; ^2^ Department of Clinical Sciences, College of Dentistry, Ajman University, Ajman, United Arab Emirates; ^3^ Center for Medical and Bio-Allied Health Sciences Research (CMBAHSR), Ajman University, Ajman, United Arab Emirates; ^4^ Research and Development, Srinivas University, Mangalore, India

**Keywords:** short chain fatty acids, oral microbiome, dental caries, periodontal health, inflammation

## Abstract

Short-chain fatty acids (SCFAs), primarily acetate (C2), propionate (C3), and butyrate (C4), are crucial microbial metabolites formed by the fermentation of dietary fibers by gut microbiota in the colon. These SCFAs, characterized by fewer than six carbon atoms, serve as an essential energy source for colonic epithelial cells and contribute approximately 10% of the body's total energy requirement. They are central to maintaining gut health through multiple mechanisms, including reinforcing intestinal barrier function, exerting anti-inflammatory effects, regulating glucose and lipid metabolism, and influencing host immune responses. Butyrate, in particular, plays a pivotal role in protecting the gut mucosa and modulating inflammatory processes. Beyond their intestinal functions, SCFAs affect systemic metabolic outcomes such as body weight regulation and appetite control by modulating the secretion of gut hormones and interacting with G-protein coupled receptors. Despite strong experimental evidence, mainly from animal models, clinical applications of SCFA modulation remain in preliminary stages, with limited translational findings in human therapeutics.In parallel, the oral microbiome also produces SCFAs, such as propionic, butyric, isobutyric, and isovaleric acids, as metabolic by-products in biofilm ecosystems like dental plaque. These acids contribute to interspecies communication, “cross-feeding” dynamics, and possibly biofilm stability or pathogenicity, especially in caries and periodontal disease. SCFAs in the oral cavity may act as signaling molecules or competitive factors, modulating microbial behavior and ecological balance within the oral niche. Collectively, these insights highlight SCFAs as integral to host-microbiota interactions, both in the gut and oral environments, with potential implications for targeted microbiome-based therapies in health and disease.

## 1 Introduction

When dietary fibers are broken down by gut bacteria in the colon, important compounds called short-chain fatty acids (SCFAs) are produced. The most common SCFAs are butyrate (C4), propionate (C3), and acetate (C2), all of which contain less than six carbon atoms. In many respects, these lipids are essential to human health. SCFAs are a significant source of energy for the cells lining the colon and provide around 10% of the body's total energy requirements. They may have an effect on weight control and metabolic health, as well as glucose homeostasis and lipid metabolism. For the overall health of the digestive system, butyrate is especially important since it ]has anti-inflammatory properties and maintains the integrity of the intestinal barrier. The body's immunological responses may be impacted by SCFAs' potential to influence gut inflammation and immune cell activity. They contribute to the production of gut hormones that regulate appetite and fullness and support a healthy metabolism. Complex carbohydrates, which evade digestion in the small intestine and are then broken down by bacteria in the colon, are the primary source of SCFAs. Foods high in dietary fiber, such as fruits, vegetables, and whole grains, promote the production of SCFAs. A diet rich in fiber is necessary to maintain proper levels of these good fats since the balance of short-chain fatty acids (SCFAs) in the gut is closely linked to immune response, metabolic health, and gut health in general.

Epidemiological studies have linked dietary fibre consumption to a decreased risk of developing diabetes, colon cancer, and cardiovascular disease. Since SCFAs have a direct impact on cell activity and function, these results may be explained. SCFA has an effect on health and illness outcomes, according to a large number of research, most of which employ animal models. However, a conclusive translation in a therapeutic setting has not yet been proven (1). The oral bacterial stream generates a variety of metabolites, including bacteriocins and other metabolic end products, to assist the species thrive in a competitive oral environment. Additionally, they generate short-chain fatty acids such as propionic, lactic, isobutyric, butyric, and isovaleric acids, which promote healthy cohabitation and interaction among the resident species. The fatty acid-secreting bacteria seen in dental caries lesions and periodontitis most likely have an impact on the biology of oral biofilms. It has been proposed that in these bacterial communities, short chain organic acids play a part in metabolic communication between cells. Oral bacteria that produce SCFAs have the ability to infiltrate the gut and affect how the gut microbiota metabolizes SCFAs. Proton-ion-dependent non-ionic diffusion and active transport facilitated by Na+- and H+-conjugated monocarboxylate transporters are the processes by which SCFAs are absorbed in the colon and cecum. SCFA concentrations in the gut vary by segment and range from 20 to 140 mM; the proximal colon usually has the highest values. Even when periodontitis is present, the colon has greater SCFA rates than the mouth cavity. However, the intestinal epithelium's cells are not harmed by this discrepancy. By analysing the morphological distinctions between the intestinal and oral mucosa, this seeming contradiction may be resolved. Intestinal cells actively absorb SCFAs, which are then partially transported and distributed through the bloodstream and partially metabolized as an energy source. On the other hand, the oral mucosa is unable to control SCFA levels, which keeps local concentrations high. According to the findings of these studies, there is “cross-feeding” between various bacterial species, and the short-chain fatty acids (SCFA) produced by oral bacteria may serve as “competitive factors in oral biofilms” and a vital source of carbon for some other oral bacteria (2).

## 2 Methodology

The literature review was conducted using PubMed, Medline, Scopus and Google Scholar databases using combinations of search terms: “Oral microbiome”, “short chain fatty acids”, “acetate”, “propionate”, “dental caries”, “periodontal health”, “inflammation”, “Oral health”. All the clinical studies, review was included between 2000 and 2025, and anything that is not relevant to oral microbiome was excluded.

### 2.1 Mechanism of production and function of short chain fatty acids

Fatty acids containing 6 or less than that are short chain fatty acids namely acetate (C2), propionate (C3), butyrate (C4), pentanoic acid (C5), and hexanoic acid (C6). Acetate, propionate, and butyrate are the major forms of short-chain fatty acids (SCFAs), which are organic acids with less than six carbon atoms that are essential for immunological regulation, metabolic balance, and gastrointestinal health. These SCFAs are produced in the colon by bacteria fermenting dietary fibers and other indigestible carbohydrates. The interconnected settings of dietary fiber and non-digestible carbohydrates, the gut lumen environment, and the gut bacteria provide the greatest framework for understanding their synthesis and function (3).

This can be elucidated under the following subheadings (a) Dietary fiber and non-digestible carbohydrates (b) Gut lumen (c) Gut microbiome.

#### 2.1.1 Dietary fiber and nondigestible carbohydrate

Dietary fibers and non-digestible carbohydrates (NDCs), which are difficult for the upper gastrointestinal system to digest and absorb, are the first ingredients in the fermentation of SCFAs. These consist of inulins, oligosaccharides, resistant starches, cellulose, hemicellulose, and pectin. These substances act as substrates for microbial fermentation once they arrive in the colon. In contrast to simple sugars, dietary fibers are not broken down by human enzymes, while being a vital source of energy for the gut flora. In particular, soluble fibers are more fermentable and strongly linked to higher generation of SCFA (4). SCFA profiles vary by fiber; for instance, resistant starch greatly raises butyrate levels, whereas inulin and oligofructose are known to create more butyrate and propionate. The kind and amount of SCFAs that are produced are also influenced by the fibre's physicochemical characteristics, including its solubility, viscosity, and fermentability. Through a variety of processes, including energy harvesting, lipid metabolism, and blood glucose regulation, this selective fermentation influences not only the microbiota's composition but also the host's physiology. Additionally, SCFAs are signalling molecules that affect insulin sensitivity, inflammation, and appetite control by interacting with GPR41 and GPR43 are examples of G-protein coupled receptors. Beyond their function as gastrointestinal tract bulking agents, dietary fibers have physiological relevance. The range of non-digestible carbohydrates (NDCs) includes not only traditional fibers like cellulose and hemicellulose but also more recent, functional carbohydrates like resistant maltodextrins, fructo-oligosaccharides (FOS), and galacto-oligosaccharides (GOS), all of which avoid small intestine digestion and reach the colon mostly undigested. These fibers are converted into SCFAs by microbiological fermentation in the colon, which then influences immunological and metabolic processes throughout the body (5). It's interesting to note that some dietary fiber types have been demonstrated to favourably develop specific microbial taxa that contain the enzyme machinery needed for fiber decomposition, such as lactobacilli and bifidobacteria. This selectivity is the foundation of the prebiotic idea, which holds that certain NDCs used in targeted dietary treatments can maximize the synthesis of SCFA by positively modulating the gut microbiome's composition and activity (6). Fibers influence stomach emptying, glucose absorption, and cholesterol metabolism in addition to acting as substrates for microbial fermentation. SCFAs produced from these fibers have hormone-like actions that impact peripheral organs. Through central pathways involving the hypothalamus, acetate, the most prevalent SCFA, may be used in skeletal muscle, adipose tissue, and other peripheral tissues, where it contributes to lipid synthesis and hunger control. Propionate primarily functions in the liver, where it helps regulate cholesterol production and gluconeogenesis. Butyrate is arguably the most physiologically active SCFA in the gut, controlling cell proliferation, apoptosis, and differentiation and playing a crucial role in preserving colonic epithelial homeostasis, despite having the lowest systemic concentration because of its preferential uptake by colonocytes (7).

#### 2.1.2 Gut lumen

Complex microbial populations flourish and perform fermentation in the intestinal lumen's special anaerobic environment. Since the proximal colon has the largest concentration of fermentable substrates, here is where the fermentation process mostly takes place. The lumen produces millimolar amounts of SCFAs, which are then taken up by colonocytes or circulated throughout the body. While acetate and propionate are more prevalent in peripheral blood, colonocytes quickly use butyrate in particular as their main energy source (8). The gut lumen's pH is crucial for controlling the makeup of microorganisms and the effectiveness of fermentation. Because SCFA synthesis reduces pH, harmful bacteria are inhibited and good anaerobes are favoured. Additionally, the ionized forms of SCFAs might affect water absorption and osmotic equilibrium, which helps to maintain stool consistency and avoid constipation. These SCFAs also control the formation of mucin and support the integrity of the mucosal barrier. Crucially, some transporters such sodium-coupled monocarboxylate transporters (SMCTs) and monocarboxylate transporters (MCTs) are involved in the lumen's absorption of SCFAs, helping to control their systemic effects and bioavailability (9). Bacteria, viruses, fungi, and archaea are all part of the gut microbiome, a dynamic and highly diverse microbial community. The phylum Firmicutes (e.g., Faecalibacterium prausnitzii, Roseburia spp.) and Bacteroidetes (e.g., Bacteroides spp.) contain the most common bacteria that produce SCFA. These species have particular enzymatic pathways that use acetyl-CoA synthesis, glycolysis, and reductive carboxylation to convert complex carbs into SCFAs. The amount and kind of SCFAs generated are significantly influenced by the variety and abundance of these microorganisms (10).

#### 2.1.3 Gut microbiome

The gut microbiome is a dynamic and highly diversified microbial population that includes viruses, fungus, bacteria, and archaea. The two most common phyla of bacteria that produce SCFA are Firmicutes (Faecalibacterium prausnitzii, Roseburia spp.) and Bacteroidetes (Bacteroides spp.). Through the processes of glycolysis, acetyl-CoA production, and reductive carboxylation, these species have particular enzyme pathways that convert complex carbs into SCFAs. Important factors influencing the amount and kind of SCFAs generated are the variety and abundance of these microorganisms (11). A key factor in controlling the makeup of microorganisms and the effectiveness of fermentation is the pH in the gut lumen. By lowering pH, SCFA synthesis promotes good anaerobes and inhibits harmful bacteria. Additionally, osmotic balance and water intake can be influenced by the ionized forms of SCFAs, which helps maintain stool consistency and avoid nausea. Additionally, by controlling the formation of mucin, these SCFAs support the integrity of the mucosal barrier. Importantly, some transporters, including as sodium-coupled monocarboxylate transporters (SMCTs) and monocarboxylate transporters (MCTs), are involved in the lumen's absorption of SCFAs. These transporters assist control the bioavailability and systemic effects of these molecules (12). By fermenting ordinarily indigestible substrates into bioactive chemicals like SCFAs, the gut microbiome functions as a metabolic organ that affects a broad range of host physiological processes. Numerous host and environmental variables, including as nutrition, age, genetics, drugs, and geographic location, affect the diversity and functional ability of this microbial population. Beneficial fermenters including Faecalibacterium prausnitzii, Roseburia intestinalis, and Akkermansia muciniphila, which effectively convert polysaccharides into SCFAs, predominate in a balanced gut microbiota. In dysbiotic conditions such as colon cancer, metabolic syndrome, and inflammatory bowel disease (IBD), these taxa are frequently reduced. In these circumstances, restoring SCFA-producing bacteria using probiotics, prebiotics, or faecal microbiota transplantation is becoming a practical treatment strategy (13). Through SCFAs, the microbiota has significant influence on host immunity in addition to food metabolism. Butyrate, for instance, affects immune cell gene expression via inhibiting histone deacetylases (HDACs). The development of regulatory T cells (Tregs), which are essential for preserving immunological tolerance and averting autoimmunity, is encouraged by this epigenetic modification. Similarly, it has been demonstrated that SCFAs affect the maturation of dendritic cells, the polarization of macrophages, and the generation of cytokines. Acetate has been linked to signalling pathways in the brain-gut axis that control behaviour and hunger through the activation of vagus nerves and the release of hormones including GLP-1 and PYY. Therefore, the SCFAs generated from the microbiota serve as a biological connection between dietary practices and immunological and metabolic responses in the body (14).

### 2.2 Interaction and role of short chain fatty acids

SCFA Biosynthesis Pathways: SCFAs are produced primarily in the colon by anaerobic fermentation of undigested dietary carbohydrates by the gut microbiota (Table 1).

**Table 1:** Major SCFAs, their precursors, and producing microbes.

SCFA	Microbial producers	Substrate	Site of production
Acetate	Bifidobacteria, Akkermansia	Soluble fiber, inulin	Proximal colon
Propionate	Bacteroides, Veillonella	Lactate, fucose	Transverse colon
Butyrate	Faecalibacterium prausnitzii, Roseburia	Resistant starch	Distal colon

#### 2.2.1 Interaction via receptors and transport

SCFAs interact with host tissues through:

• **GPCRs:** GPR41 (FFAR3), GPR43 (FFAR2), GPR109A
• **HDAC inhibition:** Especially butyrate, influencing gene expression
• **Monocarboxylate transporters (MCT1, MCT4):** Mediate SCFA absorption into colonocytes.

Histone deacetylase (HDAC) inhibition, G-protein-coupled receptors (GPCRs), and particular transporters such monocarboxylate transporters (MCTs). GPCRs like GPR41 (FFAR3), GPR43 (FFAR2), and GPR109A are activated by SCFAs, especially acetate, propionate, and butyrate. These GPCRs are expressed on a range of cells, including adipocytes, immune cells, and epithelial cells. Numerous physiological consequences, including the control of immunological responses, energy consumption, and intestinal motility, result from the activation of these receptors. For example, it has been demonstrated that SCFA-induced GPR43 activation mediates anti-inflammatory effects by regulating neutrophil recruitment and fostering the formation of regulatory T cells, whereas GPR41 affects energy consumption and sympathetic nervous system activity (15). Because SCFAs, particularly butyrate, block histone deacetylases (HDACs), gene expression is epigenetically regulated. By changing the chromatin structure, this HDAC inhibition promotes the transcription of genes that fight inflammation and preserve the integrity of the epithelium. Additionally, monocarboxylate transporters (MCT1 and MCT4) carry SCFAs into colonocytes and other host cells, facilitating their intracellular absorption and use as signaling molecules or energy sources. The ability of SCFAs to have systemic effects and the preservation of colonic epithelial health depend on this transport (16) (Flowchart 1).

**Flowchart 1:**
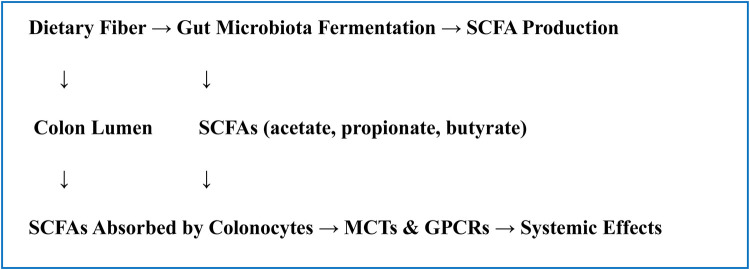
Mechanism of SCFA production and absorption.

#### 2.2.2 Functional roles of SCFAs in health

Colonocyte Energy Source: The main energy source for colonic epithelial cells is butyrate. It enhances intestinal barrier function, encourages mucin synthesis, and preserves epithelial integrity.

**Immune Modulation:** SCFAs regulate immune response by

• Enhancing **regulatory T cells (Tregs):** The commensal gut microbiota ferments food fibers to produce short-chain fatty acids (SCFAs), such as acetate, propionate, and butyrate. One of the most important of their numerous physiological roles is immunological regulation, especially the promotion of immune tolerance and homeostasis depend on regulatory T cells (Tregs). Through their modulation of Treg differentiation and function, SCFAs aid in the promotion of a balanced immune response. Both signalling pathways and epigenetic processes involving G-protein coupled receptors (GPCRs), such GPR43 and GPR109A, which are expressed on a variety of immune cells, including Tregs, are responsible for this (17, 18).

Butyrate's function in fostering Treg development has been particularly well investigated. By inhibiting histone deacetylase (HDAC), it increases chromatin accessibility and promotes the transcription of Foxp3, the Treg master regulating gene. SCFAs help transform naïve CD4+ T cells into functionally suppressive Tregs by upregulating Foxp3 expression, which reduces inflammation and autoimmune. Propionate and acetate both boost treg growth and encourage the synthesis of IL-10, an essential cytokine that reduces inflammation., which both contribute to this regulatory environment. According to Trompette et al., these alterations work together to promote a more tolerogenic immunological state, especially in the intestinal mucosa (19). SCFAs improve the suppressive capabilities of Tregs in addition to promoting their differentiation in the gut. Butyrate and propionate aid Tregs limit effector T cell responses and reduce inflammation by upregulating the production of immunosuppressive mediators including IL-10 and TGF-β. Additionally, SCFAs support the function of the gut epithelial barrier by preventing bacteria and antigens from moving around and potentially triggering immunological activation. This interaction promotes intestinal homeostasis and lessens the possibility of immunological overreaction, particularly in diseases like inflammatory bowel disease (20).

• Inhibiting **pro-inflammatory cytokines**: In many clinical scenarios, including infections, metabolic disorders, and autoimmune diseases, these cytokines play a significant part in triggering inflammation. SCFAs block nuclear factor-kappa B (NF-*κ*B) signaling, a crucial transcription factor that regulates the expression of several inflammatory genes. By preventing NF-*κ*B activation in immune cells like dendritic cells and macrophages, SCFAs lessen the inflammatory response by lowering the synthesis and release of IL-6, TNF-α, and other inflammatory mediators. SCFAs decrease inflammation through the inhibition of histone deacetylase (HDAC), namely by butyrate, which alters chromatin structure and suppresses the expression of pro-inflammatory genes. This epigenetic alteration reduces the capacity of innate immune cells to produce inflammatory cytokines in response to pathogen or danger signals. Additionally, through G-protein-coupled receptors like GPR43 and GPR109A, SCFAs can modify intracellular signalling pathways that promote anti-inflammatory phenotypes, including increased IL-10 production and macrophage polarization towards an M2-like, anti-inflammatory state (21).
• Suppressing **dendritic cell maturation:** Short-chain fatty acids (SCFAs), especially butyrate and propionate, have a significant effect on immune regulation because they prevent dendritic cells (DCs) from maturing and functionally activating, which is necessary for inducing adaptive immunological responses. Immature DCs often collect and process antigens. When they reach adulthood, they go to lymphoid tissues where they expose naïve T cells to these antigens, causing them to develop into subsets of effector T cells. During this process, pro-inflammatory cytokines including TNF-α and IL-12 are released, and costimulatory molecules (CD80, CD86, and CD40) and major histocompatibility complex class II (MHC-II) are elevated. Exposure to SCFAs alters this trajectory, particularly through butyrate's suppression of histone deacetylases (HDACs), which alters the patterns of gene expression required for full DC maturation. Because the DC phenotype expresses less costimulatory molecules and inflammatory cytokines, it becomes semi-mature or tolerogenic, reducing the likelihood of inducing strong T cell-mediated immune responses. DCs express certain G-protein-coupled receptors, including GPR43 and GPR109A, which initiate intracellular signaling cascades that further prevent DC maturation and pro-inflammatory potential. Through these receptors, SCFAs function.

In addition to decreasing the production of DC cytokines (such as IL-12p70 and IL-6), butyrate or propionate activation of these receptors improves their ability to promote the development of regulatory T cells (Tregs) rather than effector T cell subsets (22).

**Metabolic Regulation: Acetate and propionate** enter systemic circulation and modulate lipid and glucose metabolism (Table 2).

**Table 2:** Metabolism of short chain fatty acids.

Function	Acetate	Propionate	Butyrate
Lipogenesis	↑ in liver	↓ via PPARγ	Neutral
Gluconeogenesis	Weak inhibitor	Inhibits in liver	No effect
Appetite regulation	Stimulates via GPR41	Inhibits via GPR43	↓ Ghrelin

**Figure 1:**
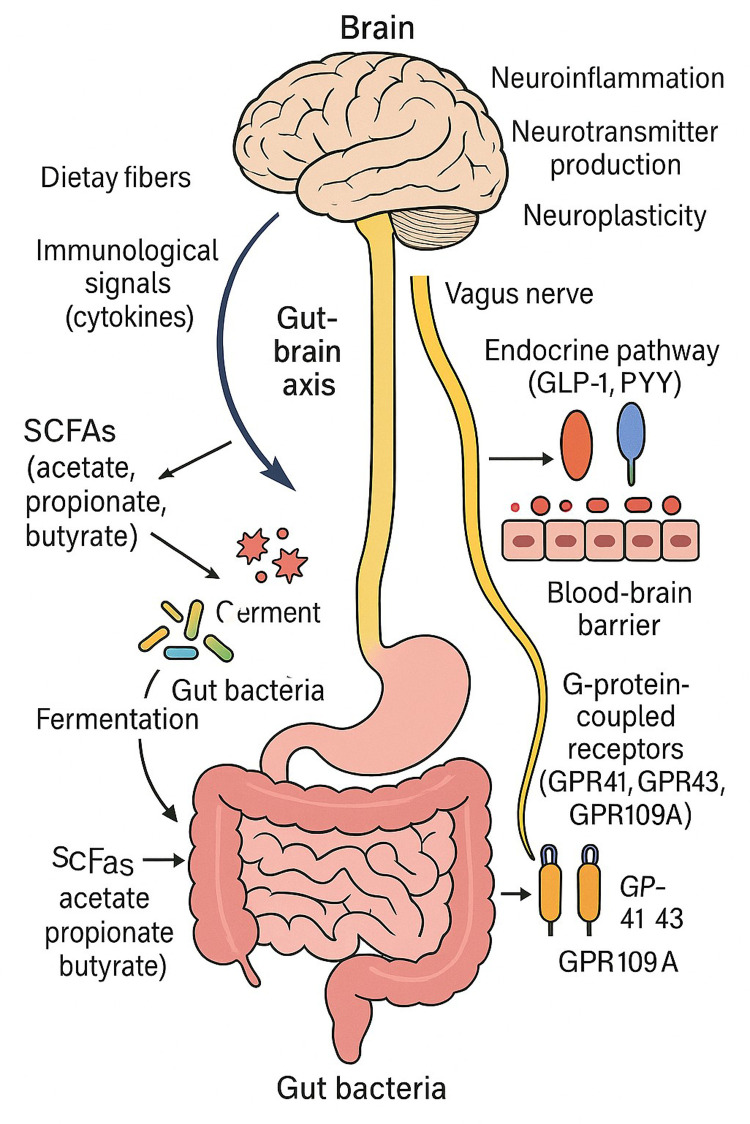
This figure illustrates the connection between gut microbes and the central nervous system via the gut-brain axis. SCFAs (acetate, propionate, and butyrate) are produced when the gut bacteria ferments food fibers. These SCFAs influence the vagus nerve, endocrine pathways (by GLP-1 and PYY), immunological signals (via cytokines), and the brain itself when they cross the blood-brain barrier. Neuroinflammation, neurotransmitter synthesis, and neuroplasticity are all impacted by SCFAs through G-protein-coupled receptors (GPR41, GPR43, and GPR109A).

**Gut-Brain Axis Influence**: By stimulating vagal afferents, controlling neurotransmitters (serotonin production), altering microglia activity, and reducing inflammation in the brain, SCFAs have an impact on the central nervous system. The gut-brain axis is a two-way communication network that links the central nervous system (CNS), enteric nervous system, and gut microbiota. Using several direct and indirect methods scfa's are becoming key mediators in this conversation (Figure 1).

Passing over the Blood-Brain Barrier (BBB): By inhibiting histone deacetylase (HDAC), SCFAs, especially butyrate, can pass through the BBB and change gene expression in the brain. By encouraging neural plasticity and lowering neuroinflammation, HDAC inhibition has neuroprotective benefits (21). Vagal Nerve Activation: SCFAs improve neuronal signalling and control mood and behaviour by modifying gut-brain connection through vagal afferents. For instance, by vagal stimulation, butyrate can cause enteroendocrine L-cells to release GLP-1 and PYY, which affect 3mood and satiety (16). Immune Modulation and Neuroinflammation By modulating systemic immune responses through GPR41, GPR43, and GPR109A receptors, SCFAs prevent neuroinflammation by lowering levels of pro-inflammatory cytokines such IL-6, TNF-α, and IL-1β (23, 50).

Acetate, propionate, and butyrate are the main short-chain fatty acids (SCFAs) produced by the colon's microbiological fermentation of food fibers. The gut-brain axis (GBA) must be regulated by these significant metabolites. Through a variety of channels, such as immunological signalling, neuroendocrine pathways, epigenetic modification, and direct neuronal communication via the vagus nerve, these metabolites affect the function of the central nervous system (CNS). Among these, butyrate functions as an inhibitor of histone deacetylase (HDAC), which affects brain gene expression and fosters neuroprotective outcomes including increased neurogenesis and synaptic plasticity (13, 24).

### 2.3 Role of short chain fatty acids in oral health

As a distinct biological niche, the mouth cavity is home to a varied microbiome that may ferment proteins and carbohydrates to produce metabolites, including SCFAs. SCFAs are created when anaerobic bacteria, including Veillonella spp., Porphyromonas gingivalis, Prevotella intermedia, and Fusobacterium nucleatum, ferment the carbohydrates and amino acids in dental plaque. The nature of the oral microbiota, dietary practices, and the existence of inflammation or illness all affect the levels of these SCFAs. These fatty acids have an impact on immunological regulation, microbial balance, and epithelial integrity as both metabolic byproducts and signalling molecules. The structure of the microbial population and the pH of the surrounding environment determine whether these metabolic byproducts are beneficial or harmful. SCFAs have been shown to interact with G-protein coupled receptors (GPR41 and GPR43) expressed on epithelial and immune cells. In the oral cavity, these interactions influence the expression of anti-inflammatory cytokines and suppress pro-inflammatory responses such as IL-6 and TNF-α production. This is particularly important in periodontal diseases where chronic inflammation leads to tissue destruction (18). Butyrate decreased inflammation via increasing the expression of regulatory T cells (Tregs) in gingival tissue. Propionate has also been shown to prevent neutrophils from infiltrating gingival crevices, which is important for controlling gingivitis early (25).

**Antimicrobial Activity and Biofilm Regulation:** Oral biofilm composition and behaviour can be influenced by SCFAs. By compromising membrane integrity and altering energy metabolism, butyrate and propionate physiologically prevent the development of pathogenic bacteria including Porphyromonas gingivalis and Fusobacterium nucleatum. According to a comparative investigation, butyrate shown more antibacterial activity but also cytotoxicity at higher doses, whereas acetate had a milder effect on oral infections. The fact that SCFAs are dual in nature emphasizes how crucial microbial balance is to preserving dental health. Increased SCFA concentrations and the periodontopathogen load in plaque biofilms are strongly correlated, suggesting that although SCFAs are advantageous in moderation, excessive synthesis might have negative effects (26, 27).

#### 2.3.1 Mechanism of SCFA production in the oral cavity

Bacterial fermentation of proteins, peptides, and monosaccharides produces SCFAs in the oral environment. The metabolic pathways, especially the Stickland reaction (amino acid fermentation) and glycolytic pathway, are similar to those found in the colon. Lactic acid bacteria like Lactobacillus and Streptococcus create lactate, which is then further broken down by anaerobes into acetate and propionate. In subgingival plaque, butyrate is mostly produced in anaerobic environments when proteolytic bacteria break down peptides from host proteins or tissue degradation. Butyrogenic bacteria thrive in periodontal pockets with low oxygen tension. SCFA's have a beneficial role in a healthy oral microbiota by: Encouraging anti-inflammatory Treg cells by stimulating immunological signalling through G-protein-coupled receptors (GPR41, GPR43). Tight junction protein expression and histone deacetylase (HDAC) suppression to improve epithelial barrier function. Promoting the salivary glands' production of antimicrobial peptides, such as cathelicidins and β-defensins. Altering the makeup of saliva to preserve the microbial balance and mouth ph. By doing these things, you may lessen inflammation, stop germs from growing, and maintain the integrity of your oral tissue (28) (Table 3).

**Table 3:** Summarizes the role of SCFA in oral health.

SCFA Production in the Oral Cavity	Anaerobic bacteria (e.g., *Veillonella*, *P. gingivalis*) ferment carbohydrates and proteins. Major SCFAs: Acetate, Propionate, Butyrate. Produced via glycolysis and Stickland fermentation.
Local Oral Effects of SCFAs	*Anti-inflammatory*: Activate GPR41/GPR43 receptors, increase Tregs, reduce IL-6 and TNF-α. *Epithelial barrier*: Boost tight junction proteins, inhibit HDAC. *Salivary glands*: Enhance cathelicidins and β-defensins. *Excess SCFAs*: Linked to cytotoxicity and increased pathogen load.
Antimicrobial & Biofilm Modulation	Butyrate and propionate disrupt *P. gingivalis*, *F. nucleatum*. Inhibit biofilm growth by disrupting membrane integrity. Acetate has mild antibacterial effects. High concentrations, especially of butyrate, may be cytotoxic.
Gut-Oral Axis Influence	SCFAs from dietary fiber fermentation by gut microbes enter circulation. Modulate immune response by increasing Tregs and reducing IL-1β, IL-6, TNF-α. Influence oral immunity systemically.
Impact on Periodontal Disease	Gut dysbiosis → leaky gut → LPS and antigens in bloodstream. Activates macrophages and neutrophils in gingiva. Leads to chronic gingival inflammation. Reduced SCFA levels impair epithelial defense.
Influence on Dental Caries	High-sugar diet → ↓ SCFAs → reduced immune modulation. Dysbiosis affects salivary secretion (IgA, mucins, AMPs). Weakened salivary defense increases risk of enamel demineralization.
Conclusion: Balance is Key	SCFAs are protective in moderate levels. Both excess and deficiency contribute to oral disease. Gut health and oral health are closely interconnected.

## 3 Discussion

**Figure 2:**
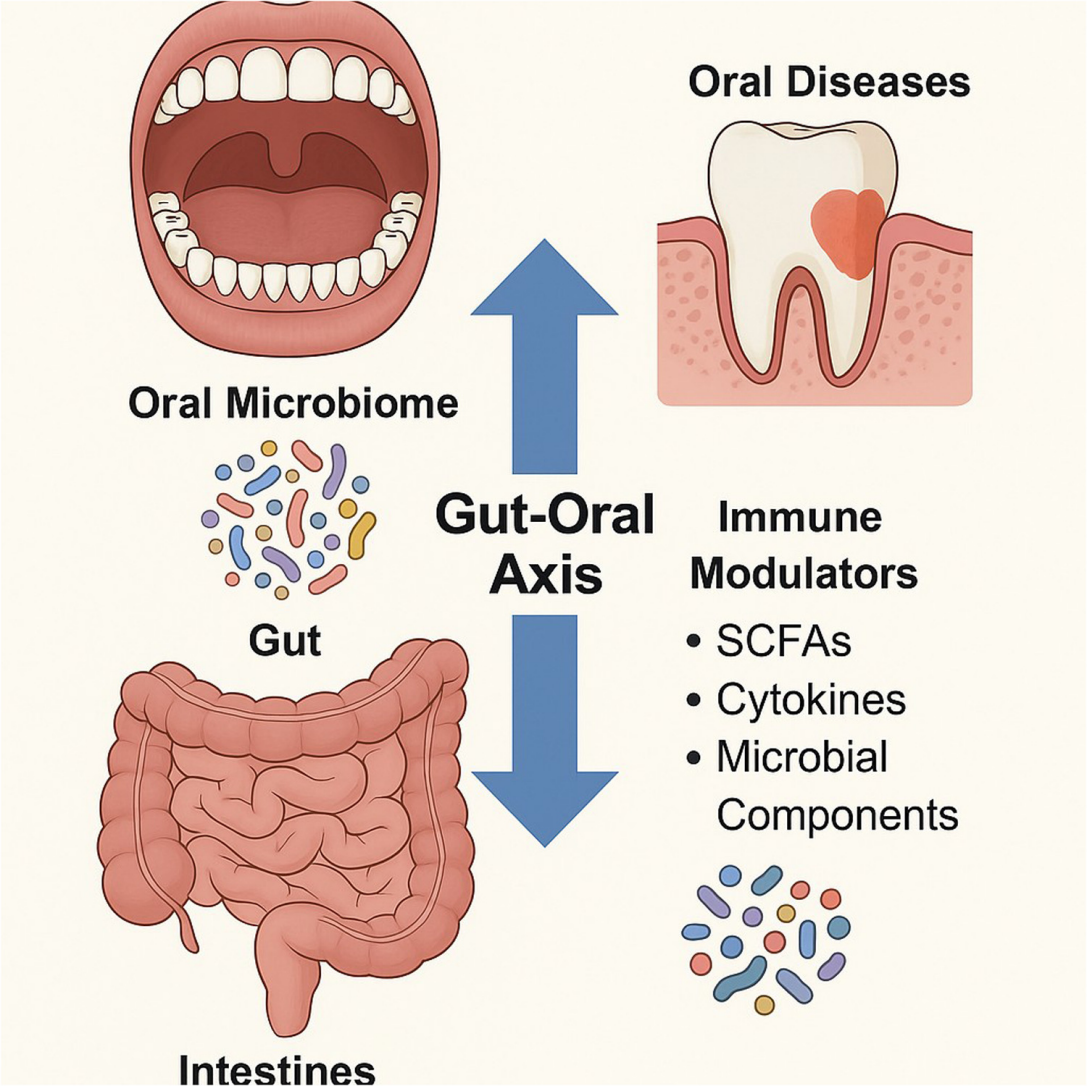
Bidirectional interaction between the gut and oral microbiomes, and highlights how gut-derived immune modulators like SCFAs, cytokines, and microbial components influence oral health and vice versa.

With more than 100 trillion bacteria, the human gut microbiome is essential for systemic and distant-site equilibrium, including dental homeostasis, in addition to gastrointestinal health. The so-called “gut-oral axis,” which links the gut and oral microbiomes, is where immunological signals and microbial metabolites affect dental health outcomes. These include short-chain fatty acids (SCFAs), particularly acetate, propionate, and butyrate, which are generated by the fermentation of dietary fibres by gut microbes and operate as systemic signalling molecules that control inflammation, mucosal immunology, and the role of the barrier between cells. The maturation of T regulatory (Treg) cells and the regulation of pro-inflammatory cytokines including IL-1β, IL-6, and TNF-α are the gut microbiota affects the host's immune response in two ways. These immune pathways have a key role in the pathophysiology of periodontal disease and tooth caries. An increased inflammatory response to plaque biofilms, for example, has been demonstrated to result from dysbiosis in the gut microbiome altering TLR (Toll-like receptor) expression on oral epithelial cells (29). Furthermore, a bidirectional interaction between the gut and oral immune milieu is suggested by the fact that individuals with inflammatory bowel disorders (IBDs), which are characterized by chronic gut dysbiosis, frequently exhibit increased prevalence and severity of periodontitis. Crucially, oral immunity can be impacted by the systemic translocation of microbial components from a leaky gut, such as peptidoglycans and lipopolysaccharides (LPS), which activate neutrophils and macrophages in the gingiva. Even with a reduced bacterial burden, this “pre-activation” renders periodontal tissues more prone to inflammation, particularly in people who are genetically predisposed. Consequently, gut microbial balance directly contributes to periodontal immune dysregulation and has effects that extend well beyond the stomach (Figure 2).

In conditions including periodontitis, dental caries, and peri-implantitis, the oral cavity has higher concentrations of short-chain fatty acids (SCFAs), such as acetate, propionate, and butyrate. In periodontal disease, anaerobic organisms such as Porphyromonas gingivalis and Fusobacterium nucleatum create SCFAs, particularly butyrate. These SCFAs cause pro-inflammatory cytokines (such as IL-1β and TNF-α), weaken the epithelial barrier, and cause gingival epithelial cells to undergo apoptosis, all of which lead to the deterioration of connective tissue and bone loss. In periodontal pockets, the local accumulation of SCFAs impairs host immunity and promotes the development of chronic inflammation.

SCFAs like acetate can reduce the pH of plaque in dental caries, resulting in an acidic environment that, in conjunction with lactic acid, promotes enamel demineralization. Furthermore, although this is still being studied, butyrate's capacity to block histone deacetylases (HDACs) in oral squamous cell carcinoma (OSCC) may result in epigenetic changes that promote malignant transformation. SCFAs exacerbate tissue damage in peri-implantitis by causing inflammation surrounding dental implants. All together, these results imply that SCFAs are important in enhancing inflammatory and destructive reactions in a range of oral illnesses, contingent on their concentration and local environment (30, 31).

### 3.1 Dental caries, gut dysbiosis, and the function of microbial metabolites

The gut microbiota can affect caries risk through systemic and metabolic pathways, even though tooth caries is primarily caused by local acid generation by cariogenic bacteria such Streptococcus mutans and Lactobacillus spp. In addition to encouraging the acidity of dental plaque, diets high in fermentable carbohydrates also alter the gut microbiota, which reduces the synthesis of SCFA and beneficial fibre-degrading species. The oral mucosa's resistance to microorganisms that cause dental cavities is indirectly compromised by decreased SCFA levels, which also impair immunological modulation and the integrity of the epithelial barrier (32). Additionally, through systemic mediators, the gut microbiome affects the makeup and function of the salivary glands. Salivary IgA, mucins, and antimicrobial peptides are secreted differently when SCFAs and other gut metabolites interact with salivary gland receptors (GPR41/43). These elements are essential for lowering bacterial adhesion, buffering plaque pH, and neutralizing acids. Gut dysbiosis impairs the salivary response, making enamel surfaces more susceptible to acid assault and demineralization (33).

### 3.2 Mechanisms and clinical data of gut microbiota and periodontal disease

A dysbiosis subgingival biofilm causes the death of supportive periodontal tissues in periodontitis, a chronic inflammatory disease. Numerous direct and indirect effects of the composition of the gut microbiota on the onset and progression of periodontal inflammation have been shown. Endotoxins and microbial antigens can move into the circulation due to decreased SCFA synthesis and increased gut permeability (also known as a “leaky gut”) caused by a dysbiotic gut microbiota. These substances can worsen the deterioration of periodontal tissue and cause systemic inflammation (34) (Table 4).

**Table 4:** Summarizes studies conducted correlating various SCFA's and oral mucosal cells (35–48).

Author(s)	Objective	Methodology	Outcome
Ueda et al. (35)	Evaluate SCFA influence on fibroblast function	Human gingival fibroblast cultures treated with SCFAs	SCFAs suppressed fibroblast proliferation and induced inflammatory cytokines
Tsuda et al. (36)	Investigate SCFA modulation of dendritic cells in oral mucosa	Mouse bone marrow-derived DCs treated with butyrate	Reduced MHC-II and co-stimulatory markers, impaired T-cell activation
Niederman et al. (37)	Evaluate metabolic profiles in gingival inflammation	GC-MS profiling of gingival tissues	SCFAs were more abundant in inflamed tissues, contributing to pathogenic biofilm
Arimatsu et al. (38)	Explore systemic inflammation from oral SCFA exposure	Mice exposed to *P. gingivalis* or butyrate orally	SCFAs induced systemic inflammation and insulin resistance
Sato et al. (39)	Examine butyrate-induced periodontitis in mice	Oral application model of butyrate; bone histology assessed	Butyrate exacerbated bone loss and inflammation
Furusawa et al. (40)	Analyze Treg induction by SCFAs in mucosa	Mouse oral mucosa treated with SCFAs	SCFAs promoted Foxp3+ Tregs via HDAC inhibition
Huang et al. (41)	Study SCFA anticancer effect on OSCC cells	OSCC cell cultures treated with sodium butyrate	Butyrate inhibited proliferation and induced apoptosis
Takahashi et al. (42)	Investigate metabolomic shifts in gingivitis	Saliva and plaque analyzed via NMR and MS	Increased SCFAs linked to early inflammation and dysbiosis
Lin et al. (43)	Study SCFA effect on barrier proteins	Oral epithelial cell lines with butyrate exposure	Butyrate reduced tight junction proteins like occludin
Sakanaka et al. (44)	Examine SCFA effects on oral immunity	ELISA-based cytokine tests in salivary epithelial cells	Butyrate increased IL-8 and TNF-α
Ishikawa et al. (45)	Evaluate SCFAs' impact on gingival regeneration	*in vitro* gingival epithelial repair assay with SCFAs	Impaired epithelial healing and growth factor downregulation
Al-Marzooq et al. (46)	Correlate diabetes, periodontitis, and SCFAs	GCF samples from diabetic vs. non-diabetic patients	Higher SCFAs in diabetics with worse periodontal status
Matsumoto et al. (47)	Study SCFAs in relation to periodontitis severity	Cross-sectional analysis of SCFA levels in GCF	SCFAs positively correlated with clinical attachment loss
Ahn et al. (48)	Explore SCFA as biomarkers for periodontitis	Saliva and GCF LC-MS metabolomics	SCFAs identified as potential non-invasive markers
Kim et al. (49)	Compare SCFA levels in peri-implantitis vs. healthy sites	SCFA quantification in peri-implant crevicular fluid	Higher SCFA levels in disease associated with tissue breakdown

## 4 Conclusion

Short-chain fatty acids (SCFAs), which are necessary for maintaining dental health, are produced when dietary fibers are broken down by the gut and mouth bacteria. These metabolites, which include butyrate, propionate, and acetate, have anti-inflammatory, antibacterial, and immunomodulatory qualities that help to preserve mucosal homeostasis, stop harmful bacteria from growing, and control host immunological responses. Changes in the synthesis of SCFAs may result from modifications in the microbiota's makeup. Oral microorganisms are naturally prevented from entering the lower gastrointestinal system by the stomach's acidic environment, the gut's alkaline environment, and the quantity of digesting enzymes. The commensal gut microbiota's ability to prevent colonization, which eradicates oral bacteria, is another crucial component. Changes in the synthesis of SCFAs may result from modifications in the microbiota's makeup. An increase in the Bacteroides/Firmicutes ratio seems to result in a relative rise in the concentration of acetate and propionate and a decrease in butyrate, despite evidence that this ratio has no effect on the overall concentration of SCFAs. Only in these situations does the.An increase in the Bacteroides/Firmicutes ratio seems to result in a relative rise in the concentration of acetate and propionate and a decrease in butyrate, despite evidence that this ratio has no effect on the overall concentration of SCFAs. Only in these instances the SCFA's may have proinflammatory effect, not when there is normal production and absorption in the intestine and oral cavity. The diagnostic and therapeutic potential of altered SCFA levels has been highlighted by their association with a number of oral illnesses, including periodontitis, dental caries, and inflammation of the oral mucosa.
